# Supplementary material for: Impact of Exosomes Released by Different Corneal Cell Types on the Wound Healing Properties of Human Corneal Epithelial Cells
Source: Int J Mol Sci. 2022 Oct 13;23(20):12201. doi: 10.3390/ijms232012201 (PMC9602716; doi:10.3390/ijms232012201)
Supplement: Supplementary file 1 [file ijms-23-12201-s001.zip › ijms-1956456-supplementary.pdf]

International Journal of *Molecular Sciences*

MDPI

Supplementary Materials

Impact of Exosomes Released by Different Corneal Cell Types on the wound healing properties of Human Corneal Epithelial Cells

Pascale Desjardins [1,2,3,4,5], Rébecca Berthiaume [1,2,3,4], Camille Couture [1,2,3,4,5], Gaëtan Le-Bel [1,2,3,4,5], Vincent Roy [1,3,5], François Gros-Louis [1,3,5], Véronique J. Moulin [1,3,5], Stéphanie Proulx [1,2,3,4], Sylvain Chemtob [6], Lucie Germain [1,3,5] and Sylvain L. Guérin [1,2,3,4,*]

1 Regenerative Medicine Division of the Centre de Recherche du CHU de Québec, Université Laval, Québec, QC G1J 1Z4, Canada
2 Centre Universitaire d'Ophtalmologie (CUO)-Recherche, Hôpital du Saint-Sacrement, 1050 chemin Ste-Foy, Québec, QC G1J 1Z4, Canada
3 Centre de Recherche en Organogénèse Expérimentale de l'Université Laval/LOEX, Hôpital Enfant-Jésus, 1401 18e Rue, Québec, QC G1V 0A6, Canada
4 Département d'Ophtalmologie, Faculté de Médecine, Université Laval, Québec, QC G1V 0A6, Canada
5 Département de Chirurgie, Faculté de Médecine, Université Laval, Québec, QC G1V 0A6, Canada
6 Département d'Ophtalmologie, Faculté de Médecine, Université de Montréal, Montréal, QC H3T 1J4, Canada
* Correspondence: sylvain.guerin@fmed.ulaval.ca

Citation: Desjardins, P.; Berthiaume, R.; Couture, C.; Le-Bel, G.; Roy, V.; Gros-Louis, F.; Moulin, V.J.; Proulx, S.; Chemtob, S.; Germain, L.; et al. Impact of Exosomes Released by Different Corneal Cell Types on the Wound Healing Properties of Human Corneal Epithelial Cells. *Int. J. Mol. Sci.* **2022**, *23*, 12201. https://doi.org/10.3390/ijms232012201

Academic Editors: Elia Ranzato and Simona Martinotti

Received: 21 September 2022
Accepted: 6 October 2022
Published: 13 October 2022

Publisher's Note: MDPI stays neutral with regard to jurisdictional claims in published maps and institutional affiliations.

Copyright: © 2022 by the authors. Submitted for possible open access publication under the terms and conditions of the Creative Commons Attribution (CC BY) license (https://creativecommons.org/licenses/by/4.0/).

Supplementary Figures

A.

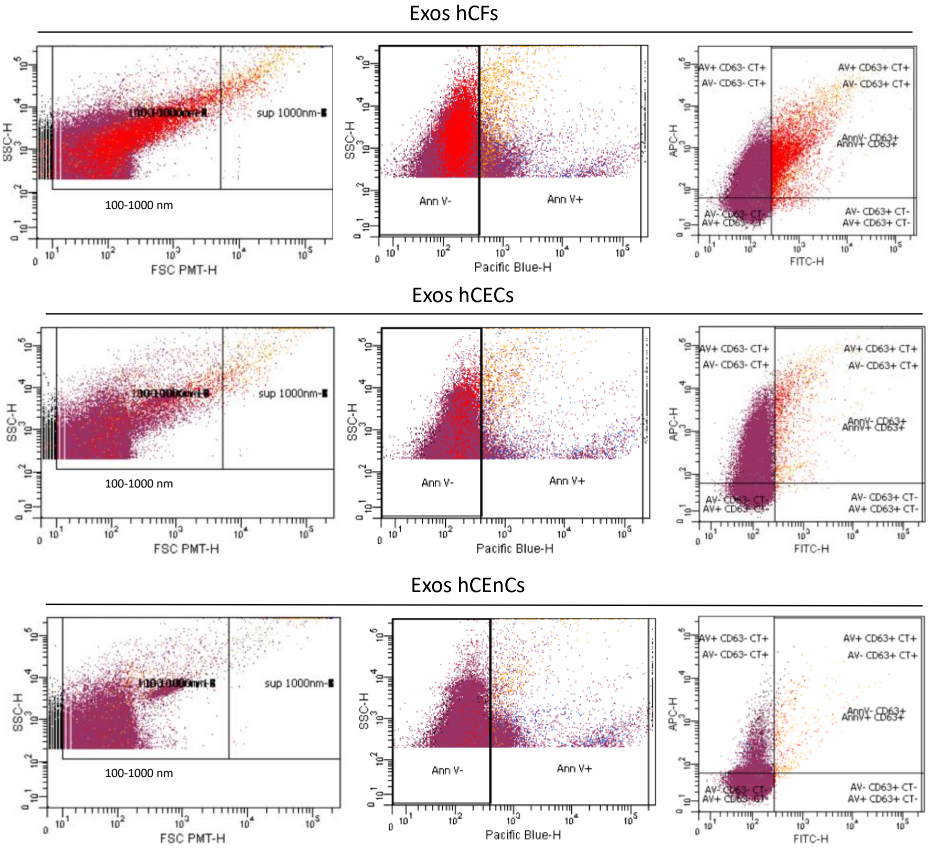

B.

		AnnexinV+ CD63- CT+	AnnexinV+ CD63+ CT+	AnnexinV+ CD63- CT-	AnnexinV+ CD63+ CT-	AnnexinV- CD63+ CT-
**# Events per mL (100-1000nm)**	Exos hCFs	216941913	22088796	208711810	5843834	22587250
	Exos hCECs	66583489	3941779	30469091	293740	924469
	Exos hCEnCs	4978057	91002	8996903	93462	159868

Supplementary Figure S1. High-sensitivity flow cytometry *(Hs-FCM)* analysis of exosomes. (A) All analyses were performed by the flow cytometry platform of the Centre de recherche du CHU de Québec-Université Laval on a BD Canto II Special Order Research Product (BD Biosciences, CA, USA) equipped with a small particle option. Acquisition was performed at low speed and quantification was assessed using a known concentration of 2µm Cy5-silica beads (Polysciences, Warrington, PA, USA) added to each tube. Fluorescent Silica particles (Kisker Biotech GmbH & Co. Steinfurt, Germany) of known dimensions (100 nm, 500 nm and 1 µm in diameter) were used for instrument set-up standardization. Samples (2.5 µl of each type of exosomes) were stained with 2µM of CellTracker Deep Red (Invitrogen, MA, USA) and labeled with an FITC anti-CD63 antibody (BD Biosciences, CA, USA) and BV421-annexin-V (BD Biosciences, CA, USA) for 30 min in the dark. To confirm the presence of microvesicles, samples were treated with 0.2% of Triton X-100 (EMD Millipore, MA, USA). At least 50,000 events were collected, and results were analyzed by DIVA 6 software. **(B)** Number of events per mL for each population of EVs detected in our three samples. AnnV and AV: annexin-V, CT: Cell Tracker.

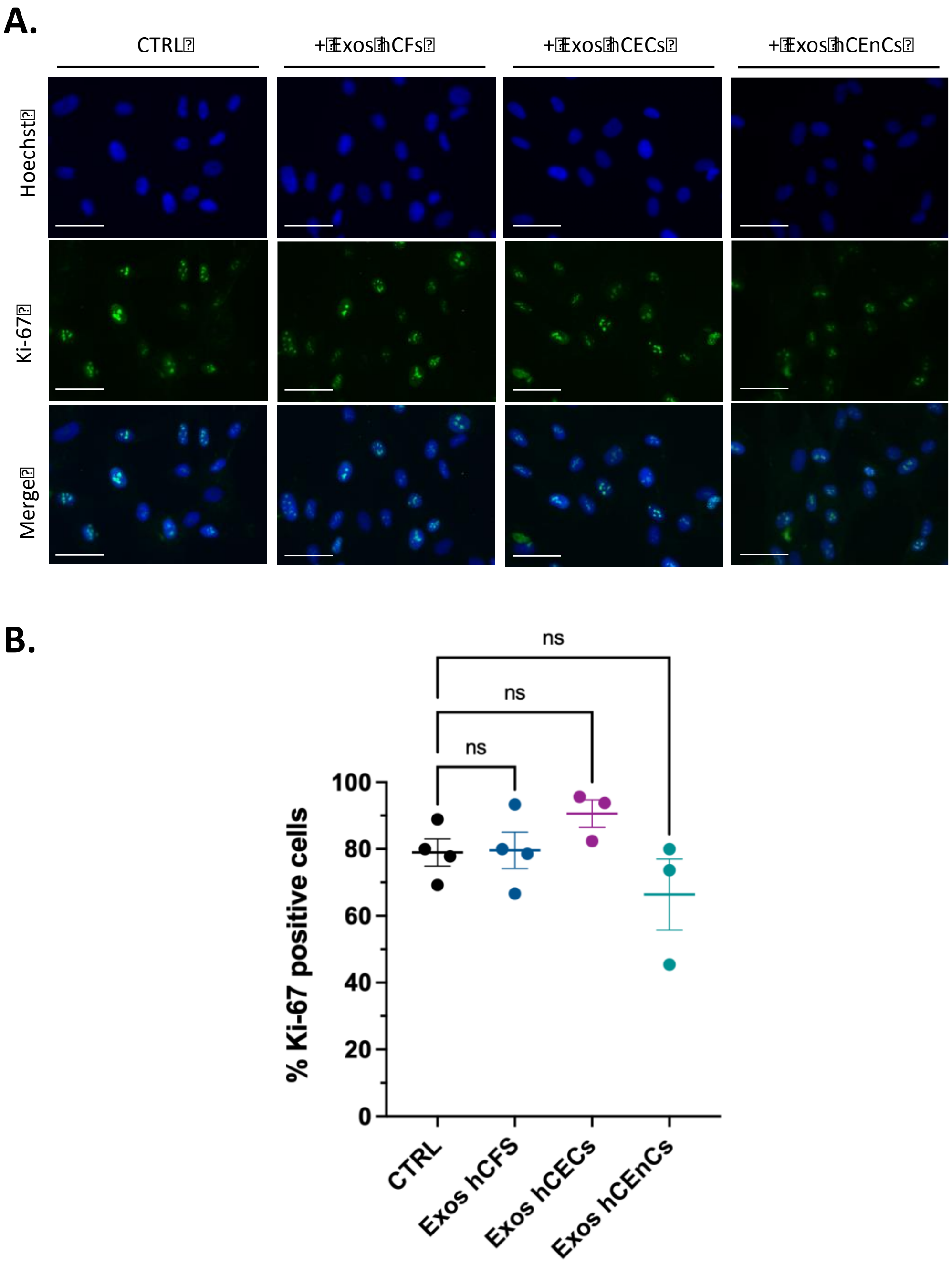

Supplementary Figure S2. Effect of exosomes from the three corneal cells types on hCFs proliferation. hCFs were cultured 48 h with 800-1000 µg exosomes isolated from conditioned medium of hCFs, hCECs and hCEnCs. HBS was used as control (CTRL). Cells were fixed and Ki-67 expression (green) was evaluated by indirect immunofluorescence. Panel **(A)** shows representative images of Ki-67 expression in hCFs. Nuclei were counterstained with Hoechst 33258 reagent (Hoechst = blue). Scale bars: 100 µm. **(B)** The number of Ki-67 positive cells in each condition was calculated and plotted on graph. The data is expressed as the mean ± SEM from three independent experiments.

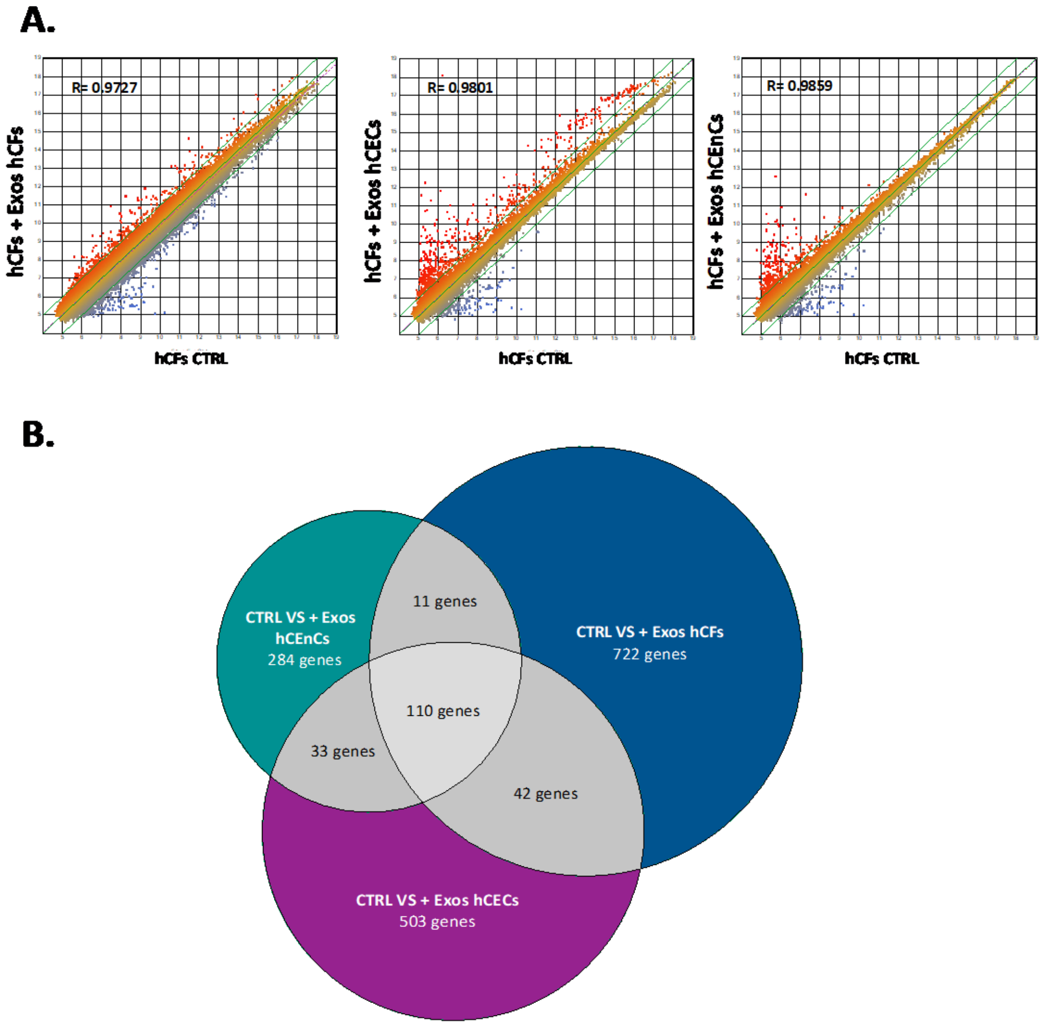

Supplementary Figure S3. Microarray analysis of the gene expression pattern of hCFs cultured in the presence of hCFs-, hCECs- and hCEnC-exosomes. (A) Scatter plot of log2 of signal intensity from 60,000 different targets covering the entire human transcriptome of hCFs + Exos hCFs (first graph), hCFs + Exos hCECs (second graph) or hCFs + Exos hCEnCs (third graph) in the y-axis as a function of hCFs CTRL (no added exosomes) in the x-axis. **(B)** Analysis of commonly regulated genes in hCFs and in our three different corneal cell types. Venn diagram depicting the number of genes differently regulated by at least a two-fold factor between hCFs CTRL and hCFs + Exos hCFs (upper right; 722 genes); genes that are specific to the hCFs + Exos hCECs condition (bottom; 503 genes) and those specific to the hCFs + Exos hCEnCs condition (upper left; 284 genes). Differently regulated genes shared between two groups are indicated at the intersections (33, 11 and 42 genes) and differently regulated genes common between the three groups are indicated in the middle (110 genes).

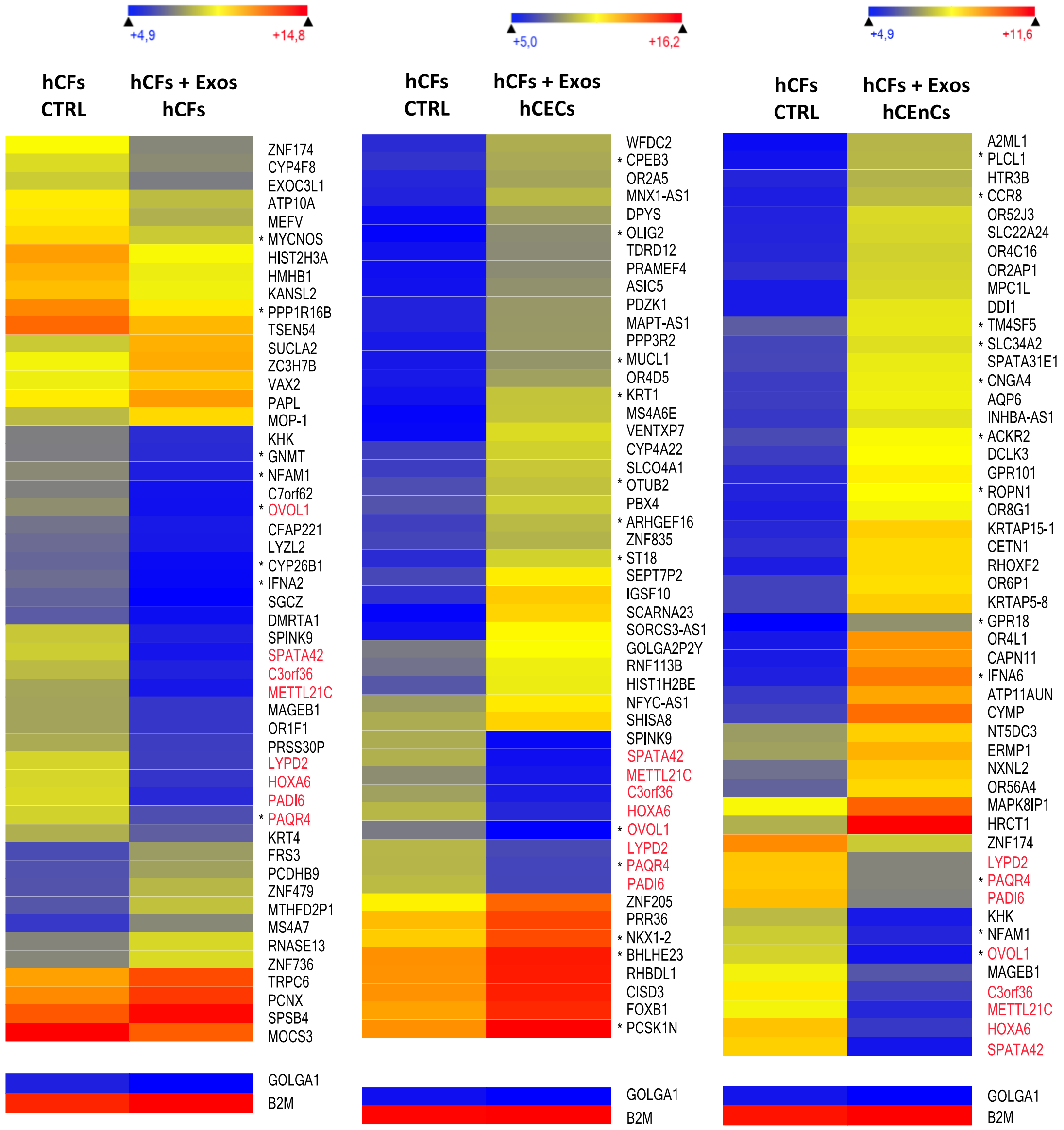

Supplementary Figure S4. Gene expression pattern of hCFs cultured in the presence of hCFs-, hCECs- or hCEnC-exosomes. Heatmap representation of the 50 most differentially regulated genes in hCFs CTRL (no added exosomes) against hCFs + Exos hCFs (first heatmap), hCFs + Exos hCECs (second heatmap) or hCFs + Exos hCEnCs (third heatmap). Gene names indicated in blue correspond to genes whose transcription are commonly downregulated in all three conditions whereas those in red are upregulated. An asterisk placed before of the gene name indicates that this gene has been associated with at least one function of interest in the IPA analysis. Microarray data for the golgin subfamily A member 1 (GOLGA1) and β2-microglobulin (B2M) housekeeping genes that are expressed, respectively, at low and very high levels in all cell types are also shown.

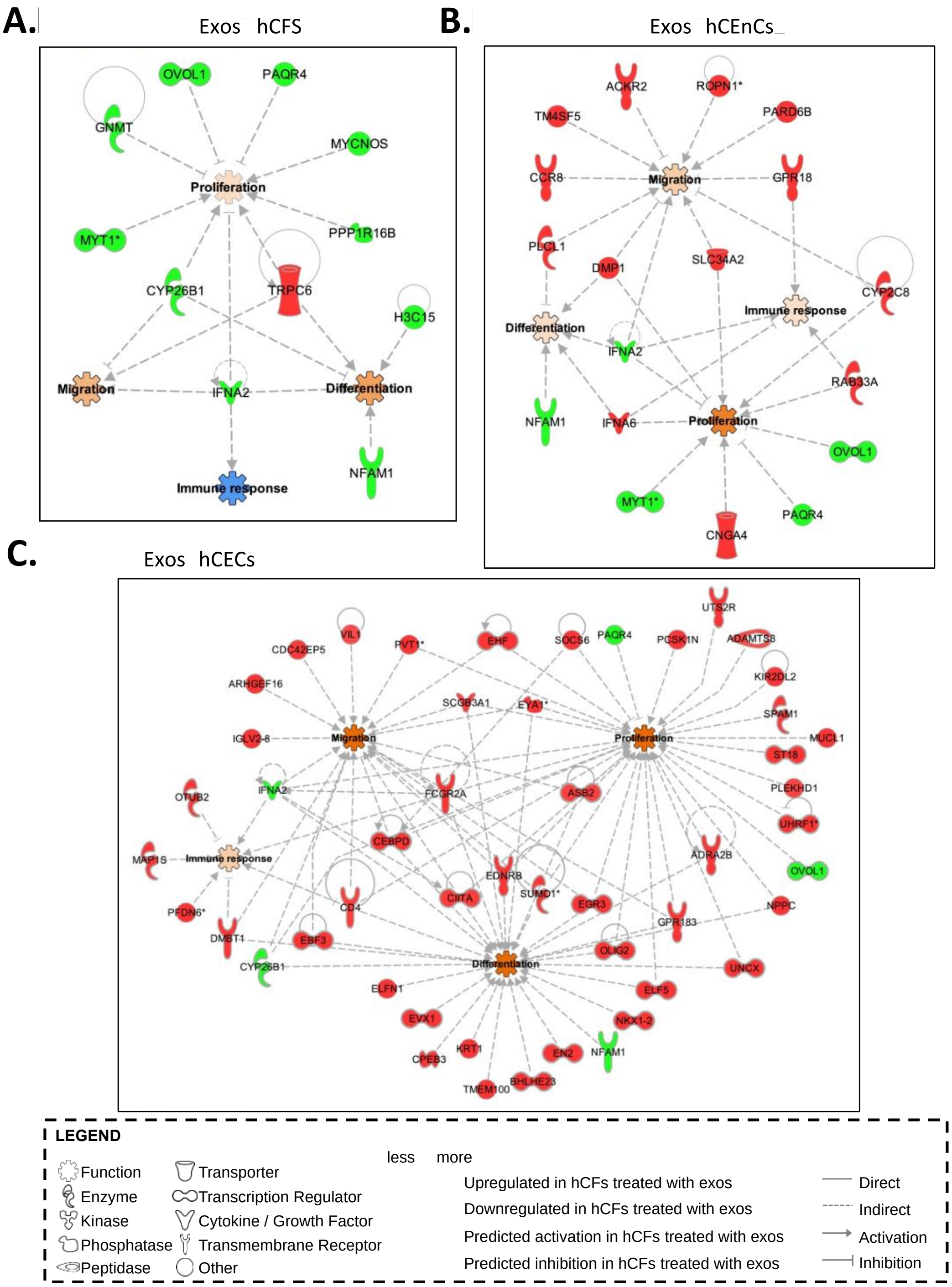

Supplementary Figure S5. Gene interaction networks modified by the addition of exosomes in hCFs. IPA generated interactome derived from differentially expressed genes in hCFs when supplemented with hCFs- **(A)**, hCEnCs- **(B)** and hCECs- **(C)** exosomes. Computationally predicted biological functions of interest (proliferation, migration and differentiation) are identified with bold labels and colored either orange or blue depending on whether they are predicted to be activated or inhibited respectively in cultures supplemented with exosomes. Differentially expressed genes present in our datasets are labelled with non-bold text and are colored either green or red depending on whether they were up- or downregulated respectively in cultures supplemented with exosomes. Lines indicate gene-gene and gene-function relationships (full lines for direct relationships and dotted lines for indirect ones) based on IPA's database. Functions are indicated in bold.

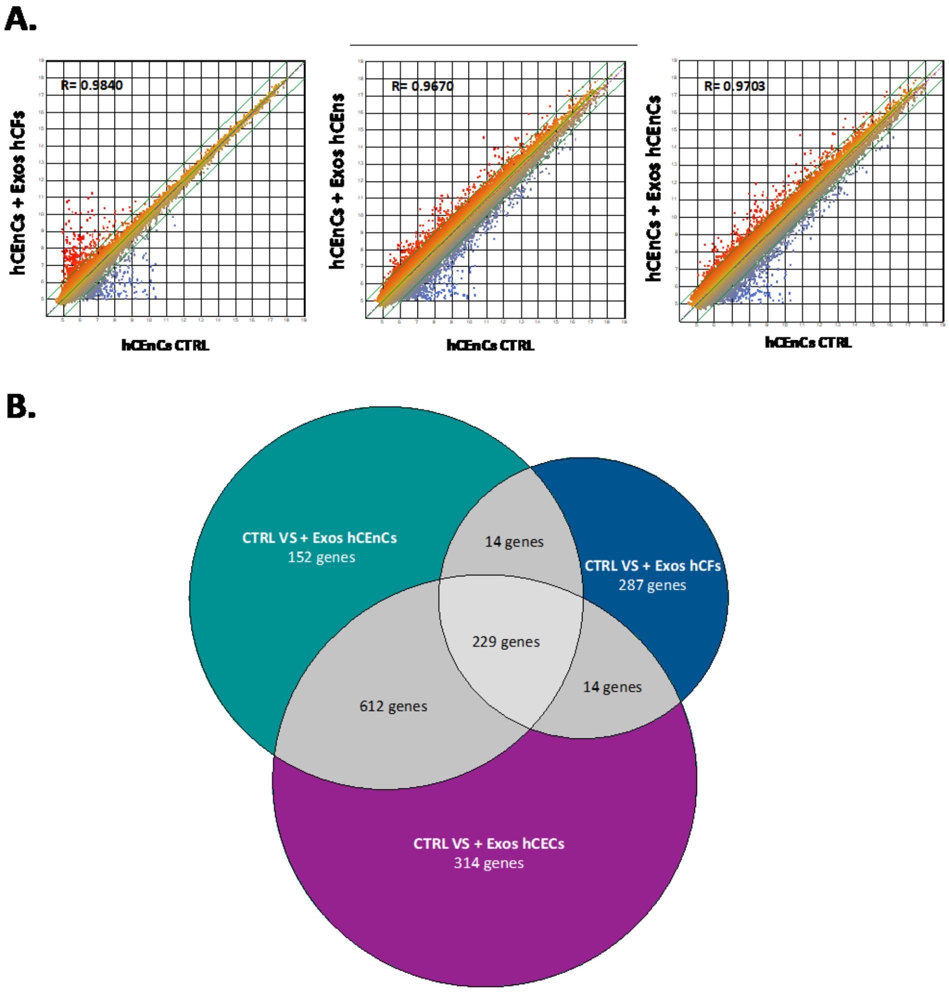

Supplementary Figure S6. Microarray analysis of the gene expression pattern of hCEnCs cultured in the presence of hCFs-, hCECs- and hCEnC-exosomes. (A) Scatter plot of log2 of signal intensity from 60,000 different targets covering the entire human transcriptome of hCEnCs + Exos hCFs (first graph), hCEnCs + Exos hCECs (second graph) or hCEnCs + Exos hCEnCs (third graph) in the y-axis as a function of hCEnCs CTRL (no added exosomes) in the x-axis. **(B)** Analysis of commonly regulated genes in hCEnCs. Venn diagram depicting the number of genes differently regulated by at least a two-fold factor between hCEnCs CTRL and hCEnCs + Exos hCFs (upper right; 287 genes) against genes that are specific to the hCEnCs + Exos hCECs condition (bottom; 314 genes) and those specific to the hCEnCs + Exos hCEnCs condition (upper left; 152 genes). Differently regulated genes shared between two groups are indicated at the intersections (612, 14 and 14 genes) and differently regulated genes common between the three groups are indicated in the middle (229 genes).

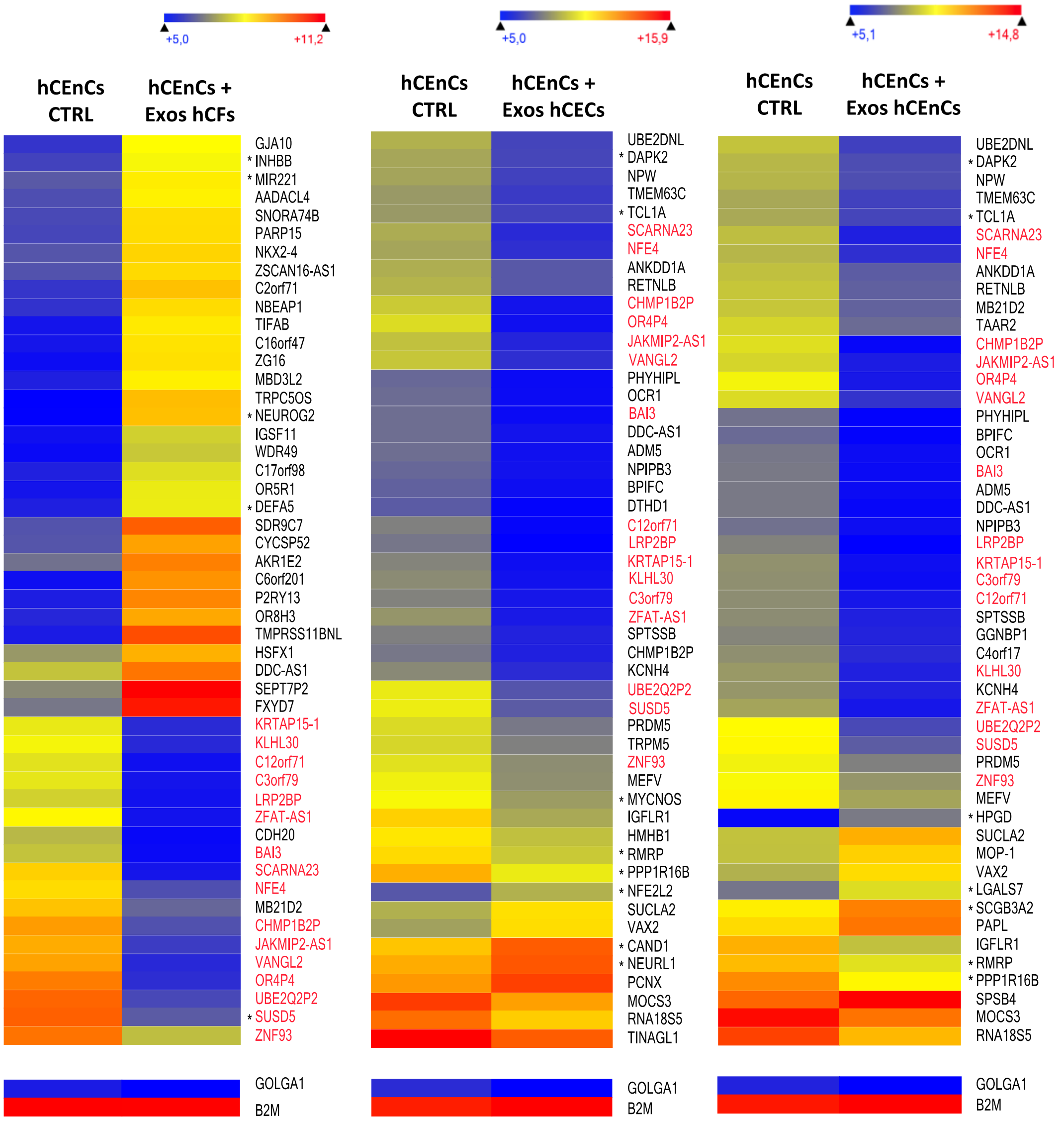

Supplementary Figure S7. Gene expression pattern of hCEnCs cultured in the presence of hCFs-, hCECs- or hCEnC-exosomes. Heatmap representation of the 50 most differentially regulated genes in hCEnCs + Exos hCFs (first heatmap), hCEnCs + Exos hCECs (second heatmap) or hCEnCs + Exos hCEnCs (third heatmap) as a function of hCEnCs CTRL (no added exosomes). Gene names indicated in blue correspond to genes whose transcription are commonly downregulated in all three conditions whereas those in red are upregulated. An asterisk placed before of the gene name indicates that this gene has been associated with at least one function of interest in the IPA analysis. Microarray data for the golgin subfamily A member 1 (GOLGA1) and β2-microglobulin (B2M) housekeeping genes that are expressed, respectively, at low and very high levels in all cell types are also shown.

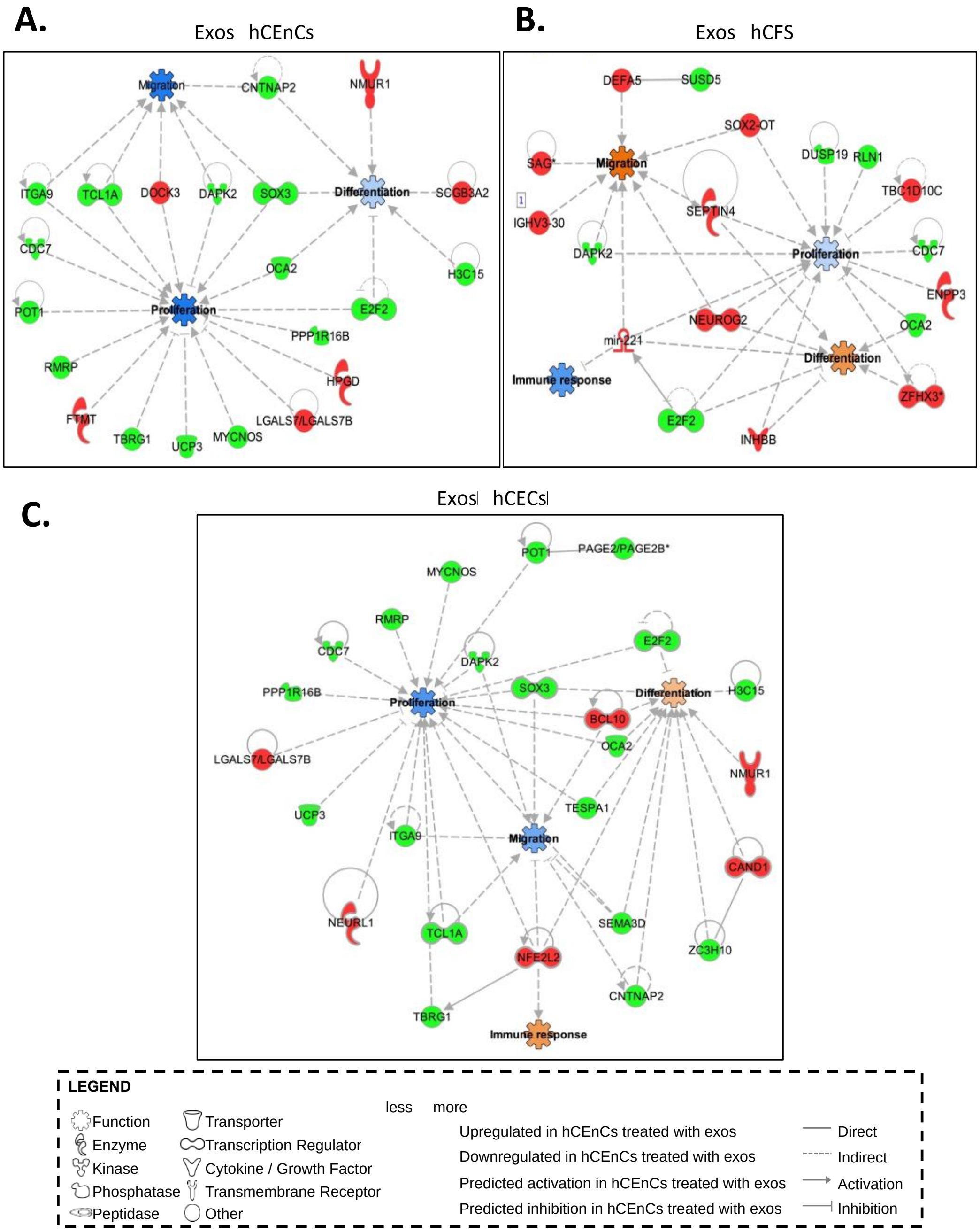

Supplementary Figure S8. Gene interaction networks modified by the addition of exosomes in hCEnCs. IPA generated interactome derived from differentially expressed genes in hCEnCs when supplemented with hCEnCs- **(A)**, hCFs- **(B)** and hCECs- **(C)** exosomes. Computationally predicted biological functions of interest (proliferation, migration and differentiation) are identified with bold labels and colored either orange or blue depending on whether they are predicted to be activated or inhibited respectively in cultures supplemented with exosomes. Differentially expressed genes present in our datasets are labelled with non-bold text and are colored either green or red depending on whether they were up- or downregulated respectively in cultures supplemented with exosomes. Lines indicate gene-gene and gene-function relationships (full lines for direct relationships and dotted lines for indirect ones) based on IPA's database. Functions are indicated in bold.

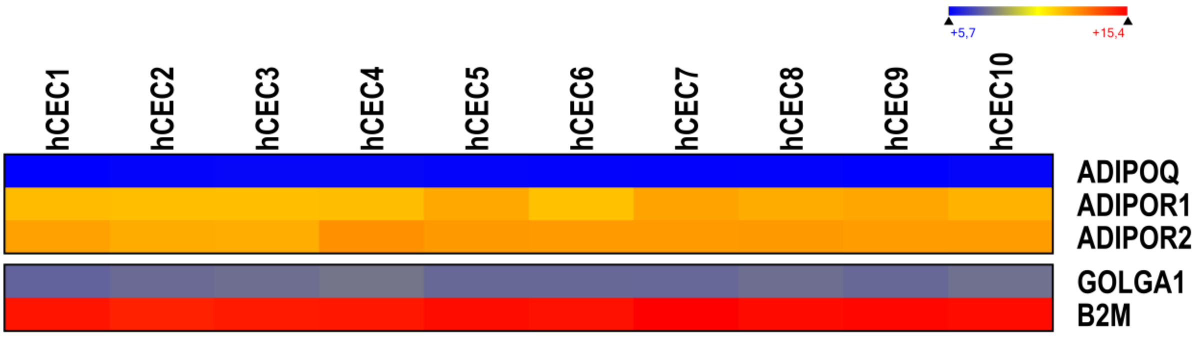

Supplementary Figure S9. Microarray analysis of adiponectin and adiponectin receptors in primary cultures of hCECs. Heatmap representation of the level of expression for the genes encoding adiponectin (ADIPOQ) or its corresponding receptors AdipoR1 (ADIPOR1) and AdipoR2 (ADIPOR2) in ten different primary cultured populations of hCECs (hCEC1 to hCEC10). Genes indicated in dark blue correspond to those whose expression is very low, whereas highly expressed genes are shown in orange/red. Microarray data for the golgin subfamily A member 1 (GOLGA1) and β2-microglobulin (B2M) housekeeping genes that are expressed, respectively, to low and very high levels in all types of cells are also shown.
